# Experimental and Mechanical Studies of Kevlar Composites

**DOI:** 10.3390/ma19183865

**Published:** 2026-09-11

**Authors:** Adam Wójcik, Danuta Miedzińska, Maciej Jan Spychała

**Affiliations:** Institute of Mechanics and Computational Engineering, Faculty of Mechanical Engineering, Military University of Technology, Kaliskiego 2 St., 00-908 Warsaw, Poland; maciej.spychala@wat.edu.pl

**Keywords:** Kevlar, aramid fibers, composites, mechanical properties, polyvinyl butyral, PVB

## Abstract

Nowadays, industries are increasingly developing advanced materials that combine high durability, sustainability, and ease of fabrication. Aramid fibers, particularly Kevlar, are widely used in engineering applications due to their excellent mechanical properties, such as high tensile strength, impact resistance, and thermal stability. At the same time, growing concerns over environmental impacts have led to increased interest in the reuse of waste materials and the development of sustainable composite systems. In this paper, we review experimental studies on Kevlar-based composites, with particular emphasis placed on composites manufactured from waste Kevlar fibers. Particular attention is given to the influence of fiber fragmentation and surface modification performed using polyvinyl butyral (PVB) on mechanical properties. We also cover material characteristics, manufacturing techniques, and the mechanical behavior of various composite systems. We primarily focus on analyzing the methodologies and research results of studies in the literature to enable future researchers to verify the quality of existing research.

## 1. Introduction

It is difficult to imagine modern civilization without polymers, composites, and hybrid materials. Scientists strive to describe materials and natural phenomena in the simplest possible terms. The beginnings of the history of plastics can be dated to 1839, when the German apothecary Simon first isolated styrene. Another breakthrough was the synthesis of nitrocellulose by Alfred Nobel. Forty-one years later, phenol–formaldehyde resins were produced under laboratory conditions. In 1947, epoxy resins were introduced into industrial production [1].

**Figure 1:**
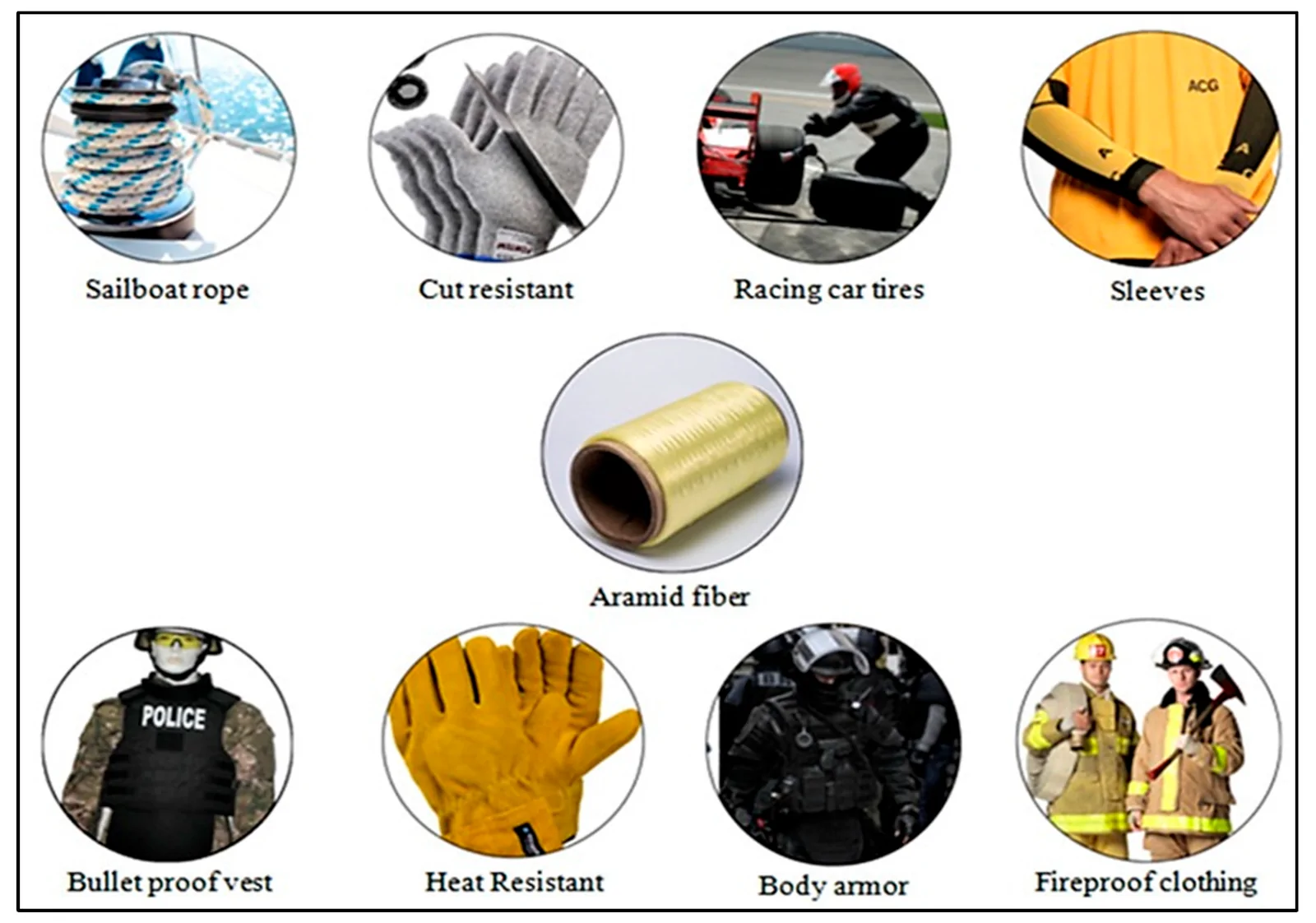
Applications of Kevlar in the industry [4].

Ongoing industrial transformation and the need for sustainable development contribute to the growing interest in the recycling and reuse of advanced composite materials. One such material is Kevlar, an aramid fiber characterized by high strength, making it suitable for protective, aerospace, and automotive applications [2]. Due to its flame resistance, high chemical resistance, and low weight, Kevlar is widely used in, among other applications, personal protective vests and helmets, aircraft components such as fuselage skins and protective panels, and the hulls of boats and special vehicles; several examples are presented in Figure 1.

A key issue determining the possibility of reusing Kevlar fibers is their fragmentation and surface modification. The literature indicates that the degree of fiber fragmentation significantly affects the stress transfer mechanism and the efficiency of fiber incorporation into the polymer matrix. Longer fibers are characterized by a greater ability to transfer loads, whereas their fragmentation improves the possibility of uniform distribution within the matrix, which, in turn, reduces local stress concentrations [3]. On the other hand, adhesion at the fiber–matrix interface remains a crucial factor determining the mechanical integrity of the composite. In this context, the application of polymer coatings, such as polyvinyl butyral (PVB), appears to be a promising solution for enhancing interfacial adhesion and improving resistance to fiber pull-out.

**Figure 2:**
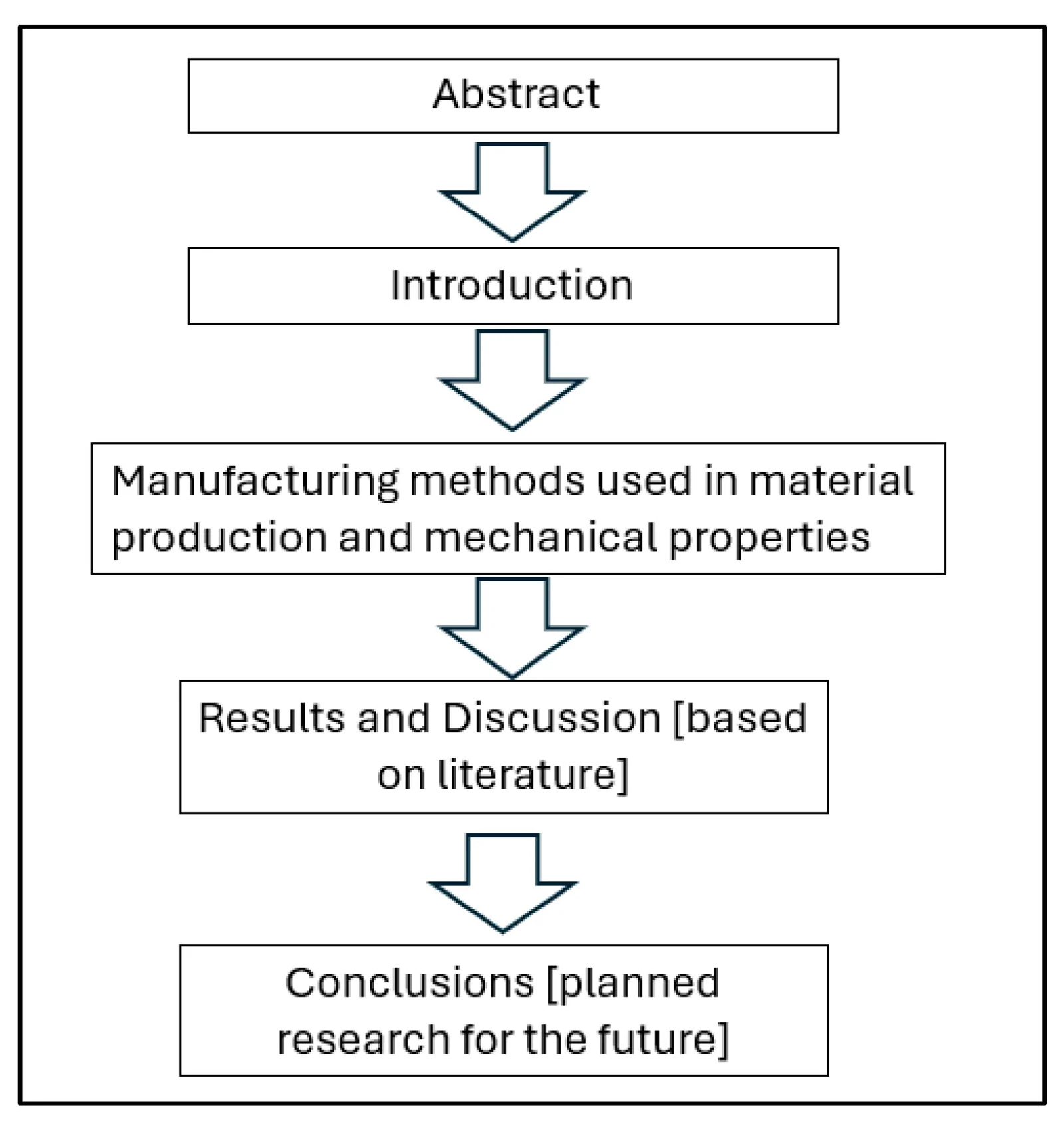
Article concept flowchart.

Despite existing studies on the recycling of fiber-reinforced composites, there are a lack of detailed investigations analyzing the simultaneous effects of the degree of Kevlar fiber fragmentation and fibers’ surface modification using PVB on the mechanical properties of polymer composites. This gap is not addressed by studies focusing solely on the technological aspects of recovering waste fibers, as illustrated in Figure 2. These fibers’ functional application in composite structures with high performance requirements is often overlooked. In response to this research gap, a broad literature review is presented in this study, the results of which we will later apply for developing a manufacturing methodology for the experimental study and modeling of recycled Kevlar–recycled PVB–Epidian composite materials for energy-intensive applications. The workflow of this study is presented as a block diagram in Figure 2 [5].

## 2. Manufacturing Methods Used in Material Production and Mechanical Properties

### 2.1. Characteristics of Hybrid Composites

**Figure 3:**
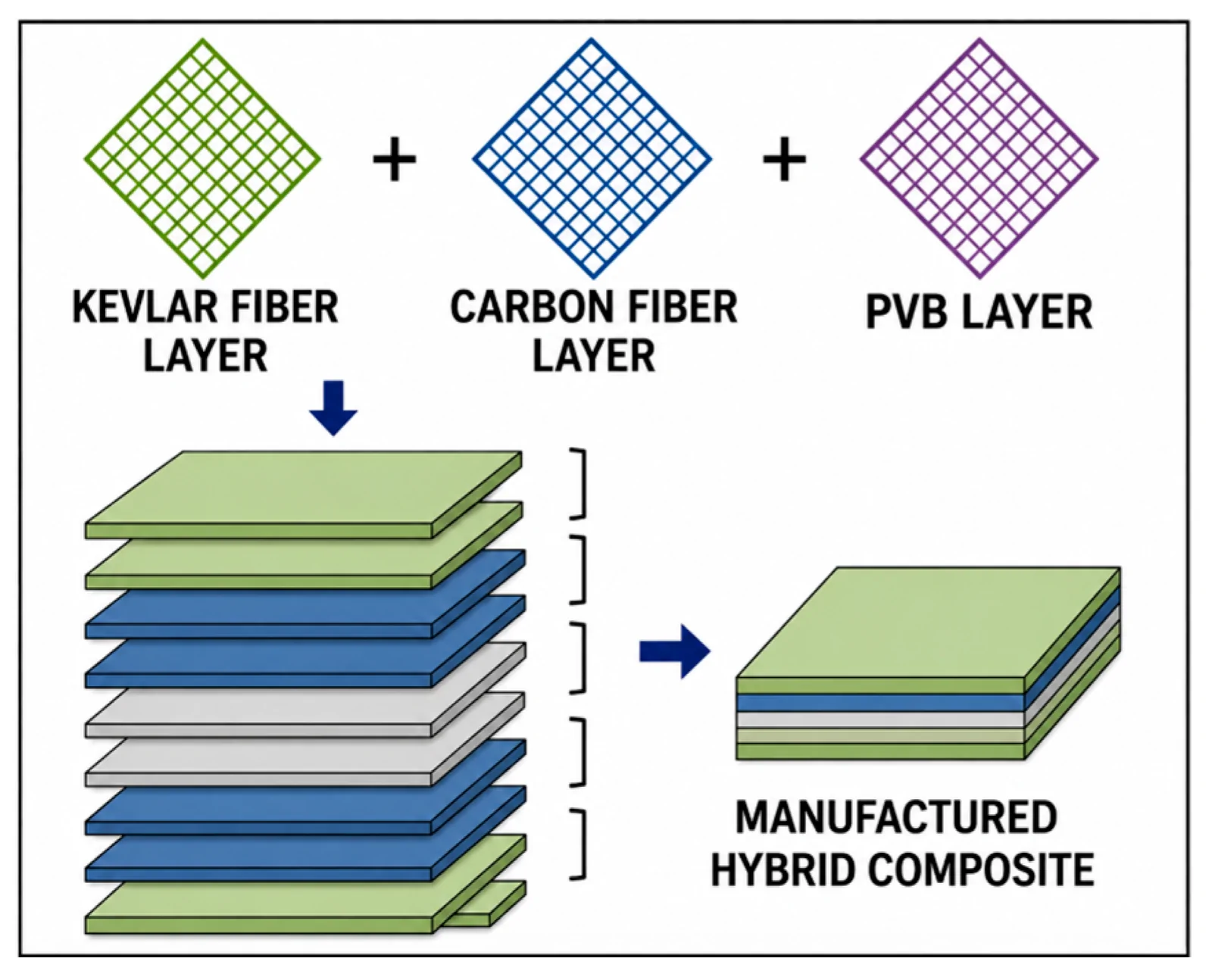
Characteristics of the layer arrangement and structure of the hybrid composite.

**Figure 4:**
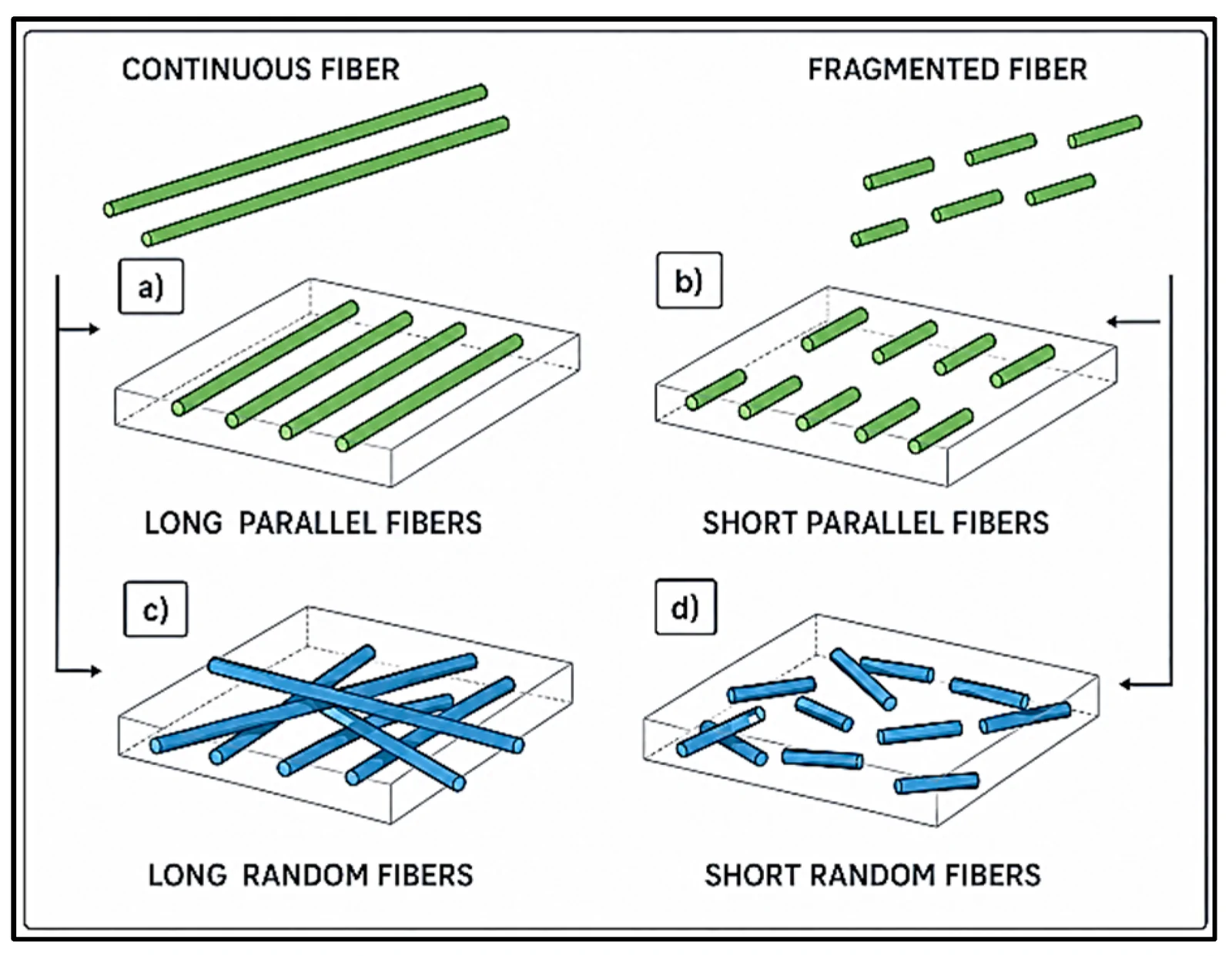
Examples of fiber dimensions in composites and the specificity of their arrangement. (**a**) continuous long fiber; (**b**) fragmented fiber; (**c**) long random fibers; (**d**) short random fibers.

One of the most significant advantages of composite technology is the ability to configure components almost arbitrarily to achieve the desired functional properties, both with respect to phase composition and geometric structure. As a result, it is possible to design hybrid materials (hybrid composites) in which both the matrix and the reinforcement consist of different types of materials, for example, combinations of carbon and aramid fibers embedded in a polymer matrix, as shown in Figure 3 [6,7]. Attention has been paid to composites in which the matrix is formed using polymeric materials, while the reinforcing phase consists of fragmented waste fibers with high mechanical strength. Such materials, known as fiber-reinforced polymer composites (including both continuous and discontinuous fiber systems), are characterized by an exceptional ability to transfer significant mechanical loads while maintaining a relatively low density, as shown in Figure 4. These properties make them particularly attractive for engineering applications, including the aerospace and automotive industries, while also enabling the reuse of production waste [8].

Appropriate reinforcement orientation selection enables very high stresses to be transferred along the fiber axis while simultaneously reducing the overall weight of the structure. This feature allows for innovative structures to be designed and the fabrication of large-scale components with complex geometries using a relatively small number of technological operations, which constitutes a major advantage of using polymer composites as structural materials. In composite materials, the degradation process is gradual and involves phenomena such as matrix microcracking, local fiber–matrix debonding, and interlaminar delamination. Although these mechanisms lead to the deterioration of material properties, they often do not result in sudden structural failure, thereby enhancing operational safety [9,10].

### 2.2. Research Methodology

**Figure 5:**
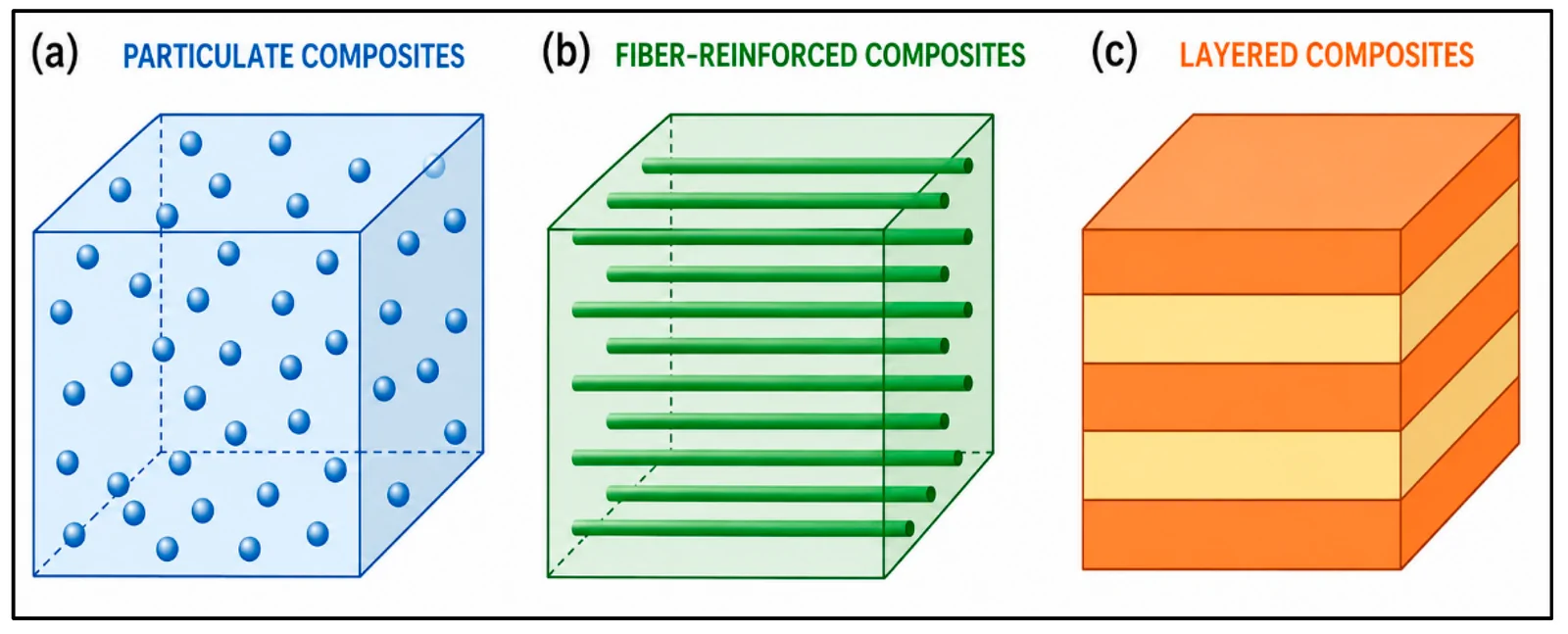
Classification of composites based on their structure.

Depending on the distribution of these phases, several main groups of composites can be distinguished, including particulate, fiber-reinforced, and layered composites (i.e., laminates), as shown in Figure 5. Each of these composite types exhibits unique properties that make them suitable for applications in various branches of industry.

### 2.3. Layered Epoxy Composites Reinforced with Aramid Fiber and Manufacturing Methods

Layered epoxy composites reinforced with aramid fibers, commonly referred to as Kevlar, constitute a significant class of advanced structural materials. Their increasing importance in aerospace, automotive, and protective applications arises from the synergistic combination of the mechanical performance of epoxy matrices with aramid reinforcement.

In this class of materials, epoxy resins act as the matrix phase, ensuring the structural integrity and continuity of the laminate, enabling efficient stress transfer between reinforcement layers, and protecting the fibers against environmental degradation. Epoxy matrices are characterized by high stiffness, strong interfacial adhesion to aramid fibers, dimensional stability, and notable chemical resistance. However, their inherently brittle fracture behavior implies that the overall mechanical response of the composite is strongly governed via the laminate architecture, as well as via the interaction mechanisms occurring at the fiber–matrix interface [11,12].

Aramid fibers are characterized by very high tensile strength, low density, and exceptional resistance to impact loading. Their molecular structure, based on strong aromatic bonds, provides high resistance to crack propagation and significant capacity for energy absorption.

**Figure 6:**
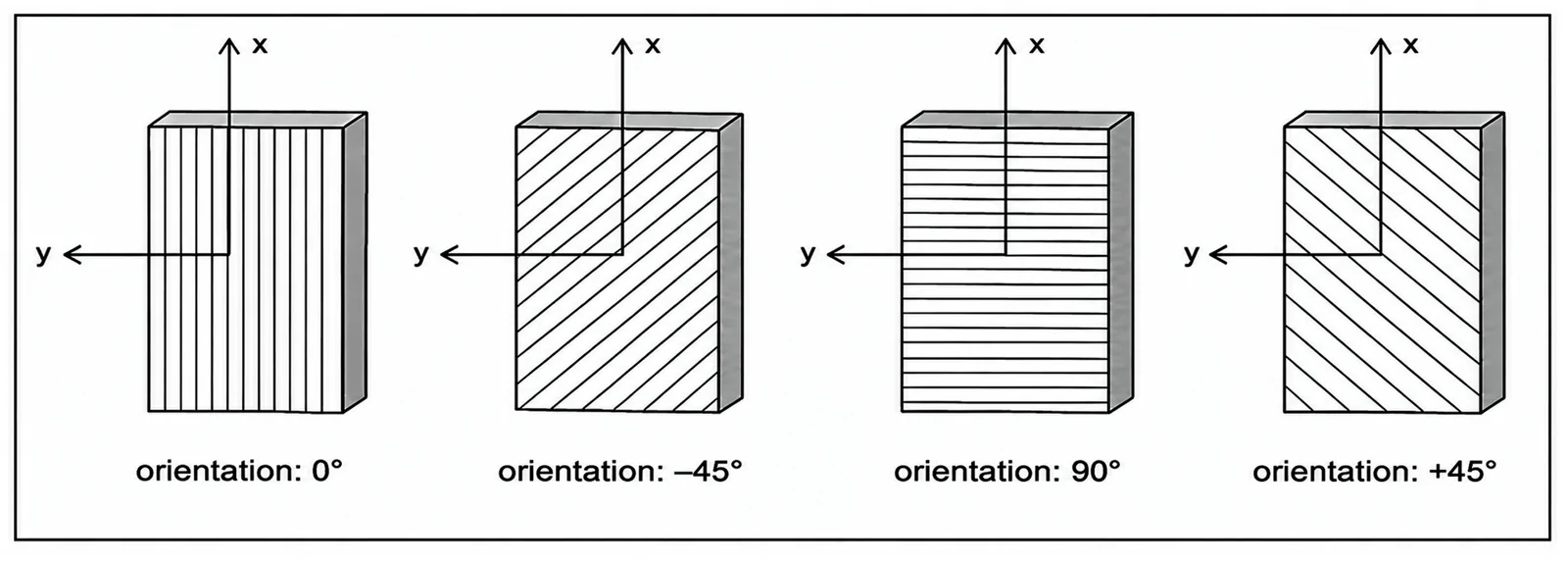
Variants of the angular orientation of fiber arrangement in reinforcement.

The layered structure of epoxy–aramid composites enables the precise tailoring of their mechanical properties through the appropriate selection of the number of layers, fiber orientation, stacking sequence, and dimensions in accordance with those specified in testing standards for composites. Multilayer laminates with varied reinforcement orientations, such as 0°, ±45°, and 90° configurations (seven samples for each variant), are employed, allowing for load transfer in multiple directions, as shown in Figure 6. Such structures exhibit pronounced anisotropy in mechanical properties, necessitating the use of advanced numerical analysis methods in the design process [13].

Epoxy resin is the most commonly used matrix material in the manufacturing of composites reinforced with Kevlar fibers. It exhibits favorable mechanical and chemical properties, including high hardness, increased thermal resistance, and water resistance. Compared to vinyl ester and polyester resins, epoxy resins demonstrate significantly lower shrinkage and do not emit harmful volatile substances during curing. Furthermore, they have excellent adhesive properties, which contribute to effective bonding with the reinforcing phase.

The manufacturing technology used for producing composite materials constitutes a key factor determining their mechanical properties, service durability, and reliability in structural applications. Regardless of the reinforcement and matrix used, the processing method governs the degree of fiber impregnation, structural homogeneity, the porosity content, and the quality of fiber–matrix interfacial bonding.

**Figure 7:**
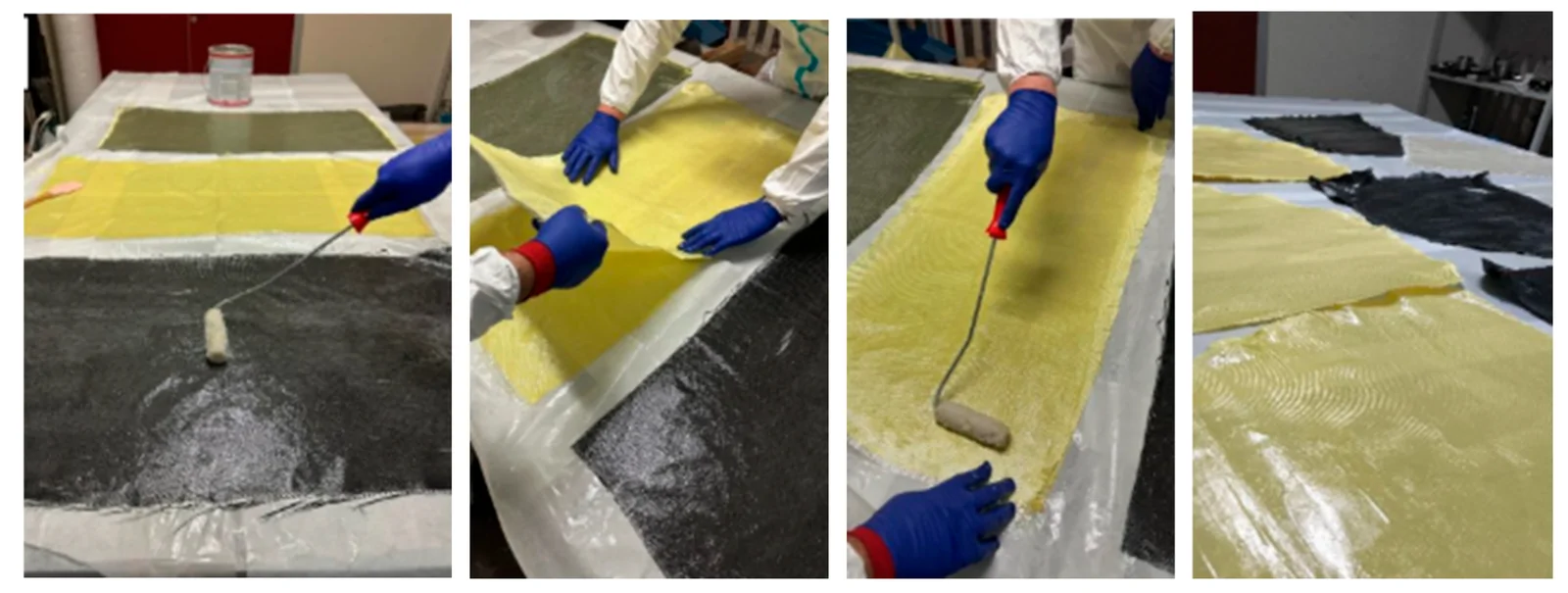
Manual composite production method [15].

As reported in [14], even minor variations in processing parameters may lead to significant differences in the strength and stiffness of polymer composites. Therefore, the selection of an appropriate manufacturing method should be closely aligned with the structural requirements and intended service conditions of the material. One of the most widely used and, at the same time, simplest manufacturing techniques for polymer composites is hand lay-up, as illustrated in Figure 7.

This method involves the sequential placement of reinforcement layers within a mold, followed by the manual application of liquid resin using brushes or rollers. Hand lay-up is primarily employed in single-unit and prototype production, as well as in the fabrication of large-scale components such as boat hulls or enclosure structures. Its main advantages include low investment costs and the flexibility to adapt the process to suit complex geometries. However, a significant limitation of this technique is the difficulty in achieving consistent quality, as confirmed by Hodgkinson’s study, which indicated considerable variability in porosity content and the occurrence of locally unimpregnated reinforcement regions [16].

To improve the quality of laminates produced via hand lay-up, vacuum bagging is often applied. This technique involves enclosing the arranged laminate stack within a vacuum bag and generating reduced pressure. The application of a vacuum enables the effective removal of entrapped air and excess resin, resulting in an increased fiber volume fraction and reduced porosity, as shown in Figure 7. As demonstrated in [17], the use of vacuum bagging can enhance tensile and flexural strength by up to several percentage points compared to conventional hand lay-up. This method is widely used in the aerospace and energy industries, particularly in the production of components with moderate mechanical performance requirements.

### 2.4. The Potential Use and Recycling of Kevlar Waste Materials and PVB

Increasing environmental awareness among consumers has led to growing demand for environmentally sustainable materials across numerous industrial sectors, including the personal protective equipment (PPE) sector. In response to these trends, manufacturers are actively developing more sustainable production technologies, as well as implementing recycling strategies for Kevlar-based products.

This transition is driven not only by the need to mitigate environmental impact but also by economic considerations. The integration of sustainability-oriented practices into material design and manufacturing processes creates new opportunities for innovation and market differentiation. Consequently, enterprises that effectively incorporate sustainable development principles into their operational strategies may achieve a significant competitive advantage in the evolving market landscape [18].

The recycling of Kevlar constitutes another important pathway for achieving market sustainability. Traditionally, Kevlar-based products have been difficult to recycle due to their complex chemical structures. However, recent advances in chemical recycling technologies have created new opportunities for the recovery and reuse of Kevlar fibers.

By developing methods for depolymerizing end-of-life Kevlar into its constituent monomers, manufacturers can establish closed-loop systems that minimize waste generation and maximize resource efficiency. Such approaches not only extend the lifecycle of Kevlar-based products but also reduce the demand for virgin raw materials, thereby contributing to environmental sustainability.

As industry transitions toward more sustainable practices, collaboration among stakeholders is becoming increasingly significant. Manufacturers, researchers, and policymakers must work together to establish standards and regulations that promote sustainable production techniques. By fostering partnerships and facilitating knowledge exchange, the market for Kevlar can accelerate the implementation of innovative practices beneficial for both the environment and the economy.

Waste Kevlar, generated either through recycling processes or via the intentional precipitation of aramid fibers from polymer solutions, constitutes an attractive precursor for further structural and functional modifications. Fibers obtained using these methods generally exhibit a lower degree of macromolecular chain ordering and inferior mechanical properties compared to conventionally spun fibers. Consequently, a key research challenge lies in the development of effective modification and reinforcement strategies for waste Kevlar fibers, enabling their reuse in advanced engineering applications [19].

Fibers derived from waste Kevlar are characterized by a specific internal structure resulting from both the original chemical architectures of aramids and the mechanical and thermal processes to which the material is subjected during service and recycling. In the case of virgin fibers, the high degree of consistent molecular chain orientation along the fiber axis, combined with a significant fraction of the crystalline phase, is responsible for their exceptional tensile strength and high modulus of elasticity.

However, processes leading to the formation of fibers from waste Kevlar, such as mechanical fragmentation, cutting, grinding, and repeated loading cycles during use, result in the partial disruption of the original ordered structure. Consequently, reductions in the degree of crystalline and the orientation of macromolecules relative to the fiber axis are observed. The structure of such fibers is distinctly semi-crystalline, with a significantly higher proportion of the amorphous phase compared to virgin fibers [19]. Polyvinyl butyral (PVB) is a thermoplastic polymer produced via the acetalization reaction of polyvinyl alcohol with butyraldehyde. Its chemical structure consists of vinyl segments, hydroxyl groups, and butyral moieties, which collectively generate a distinctive combination of mechanical, adhesive, and optical properties [20].

**Figure 8:**
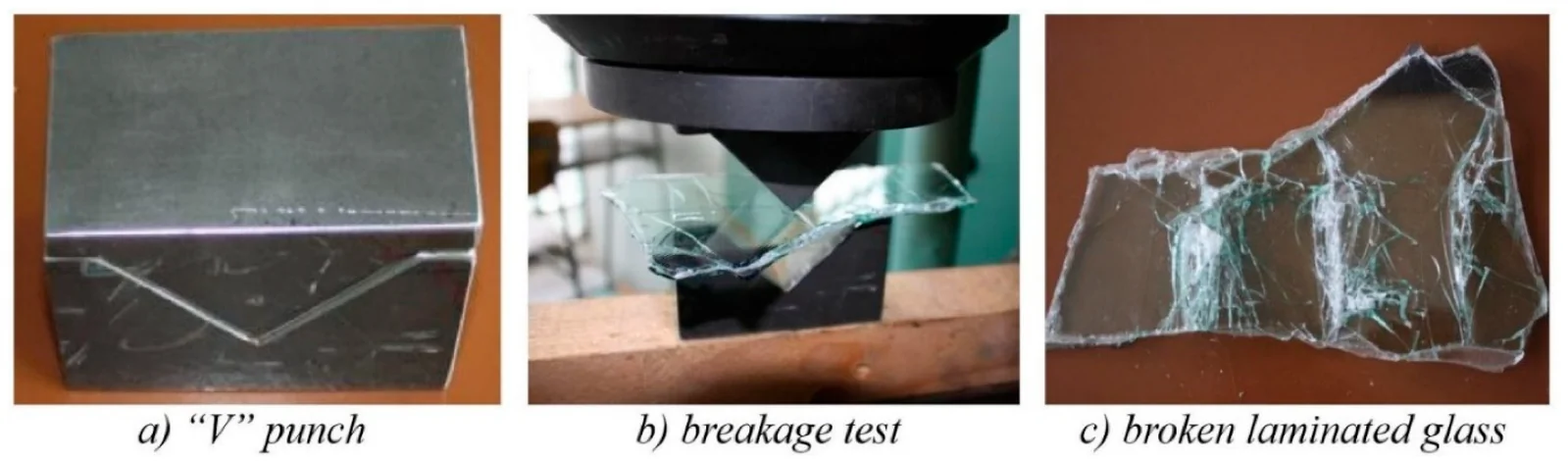
Example of laminated recycling process by glass breaking [21].

PVB is characterized by high optical transparency, considerable flexibility, excellent adhesion to glass and metallic substrates, and a significant capacity for impact energy absorption. Owing to these properties, it is extensively applied in the automotive and construction industries as an interlayer material in laminated safety glass. In such applications, PVB ensures the structural integrity of the glass assembly after fracture and contributes to enhanced impact resistance, as shown in Figure 8.

According to the literature, polyvinyl butyral (PVB) is characterized by a relatively low modulus of elasticity compared to conventional structural polymers, while exhibiting a high capacity for both elastic and viscoelastic deformation. The Young modulus of PVB at room temperature typically ranges from several tens of to several hundred megapascals and is strongly dependent on temperature, as well as the strain rate. The material demonstrates pronounced damping properties and a significant ability to dissipate energy, which is vital in protective applications. Its tensile strength generally ranges from several to several tens of megapascals, while the elongation at break may exceed 200–300%, indicating high ductility [22].

The physicochemical properties of PVB, such as resistance to environmental factors, ultraviolet radiation, and moisture, are, in industrial practice, modified through the incorporation of plasticizers, stabilizers, and other additives. PVB films used in laminated glass contain substantial amounts of plasticizers, which reduce the glass transition temperature and enhance material flexibility. The typical glass transition temperature of PVB lies is approximately 20–40 °C, indicating that under service conditions, the material often operates within the transition region between the glassy and highly elastic states, exhibiting strongly viscoelastic behavior. This characteristic directly influences its ability to absorb impact energy and damp vibrations in multilayer structures.

Polyvinyl butyral waste is primarily generated through recycling laminated glass, where the PVB interlayer is separated from the glass in the form of films or fragmented material. Such waste is characterized by significant heterogeneity, which results from the presence of glass residues, organic contaminants, adhesive remnants, and variable plasticizer content.

In summary, in the context of research on new composite materials, PVB waste can serve as a flexible polymer matrix or as a component modifying the mechanical properties of multiphase systems. Its high deformability, strong adhesion to other materials, and damping characteristics make it particularly attractive for applications requiring energy absorption, vibration reduction, and enhanced resistance to impact loading. The incorporation of PVB waste into composite structures is also consistent with the principles of the circular economy, as it enables reduced waste streams and the rational utilization of polymer resources, while simultaneously contributing to the development of materials with novel and functionally advantageous properties.

### 2.5. Mechanical Properties of Kevlar and PVB Composites

Mechanical properties constitute the primary criterion for evaluating the quality and suitability of composite materials in structural applications. For epoxy composites reinforced with aramid fibers, such as Kevlar, particular importance is attributed to parameters determining load-bearing capacity, stiffness, and resistance to mechanical damage. The most significant of these include tensile and flexural strength, compressive and shear strength, and impact resistance, delamination resistance, and the ability to transfer structural loads during service. The investigation of mechanical properties provides essential data required both at the design stage and in the numerical modeling and structural validation of composite materials [23].

**Figure 9:**
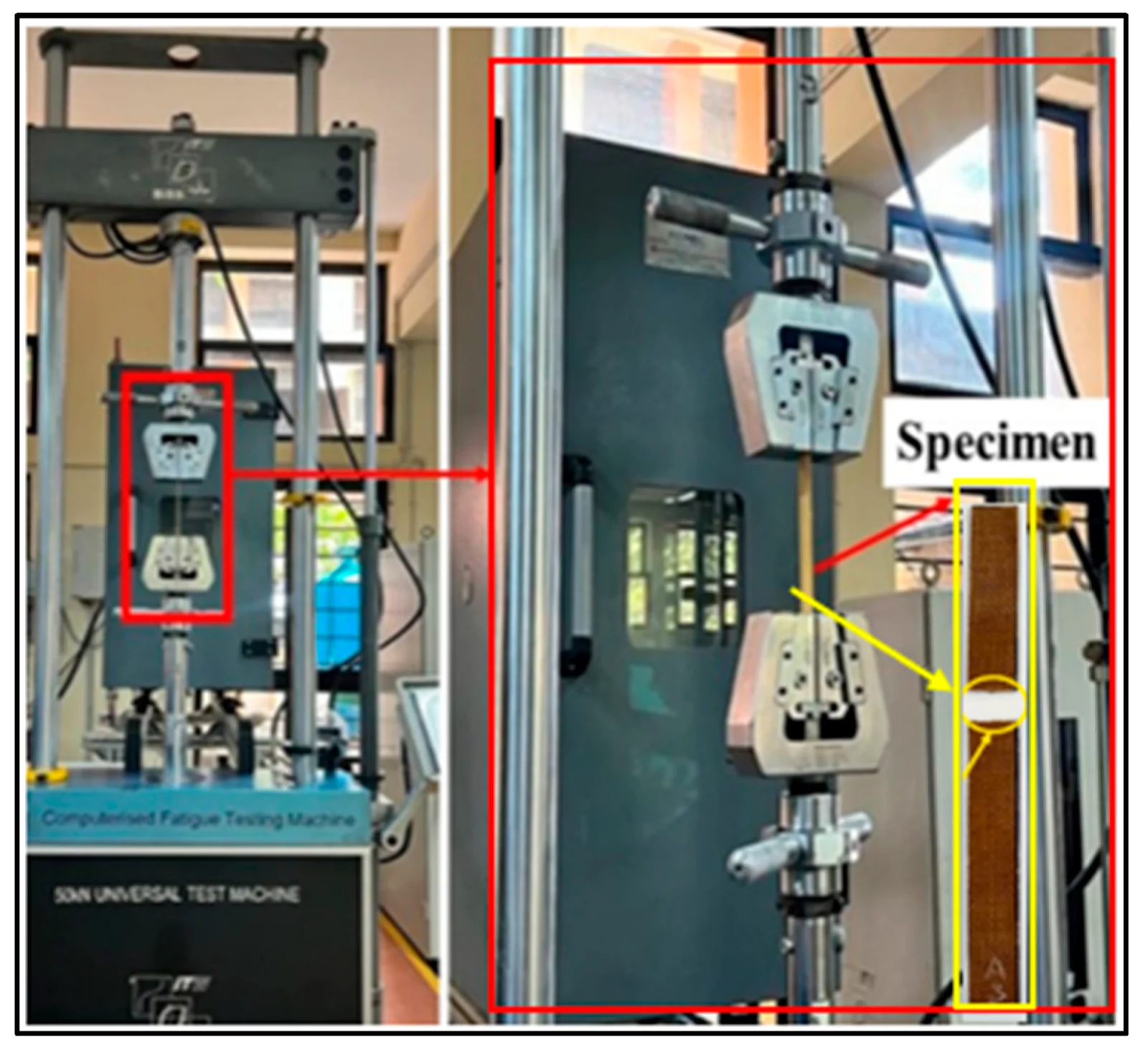
Test setup used for conducting the tensile test with the tested specimen.

**Figure 10:**
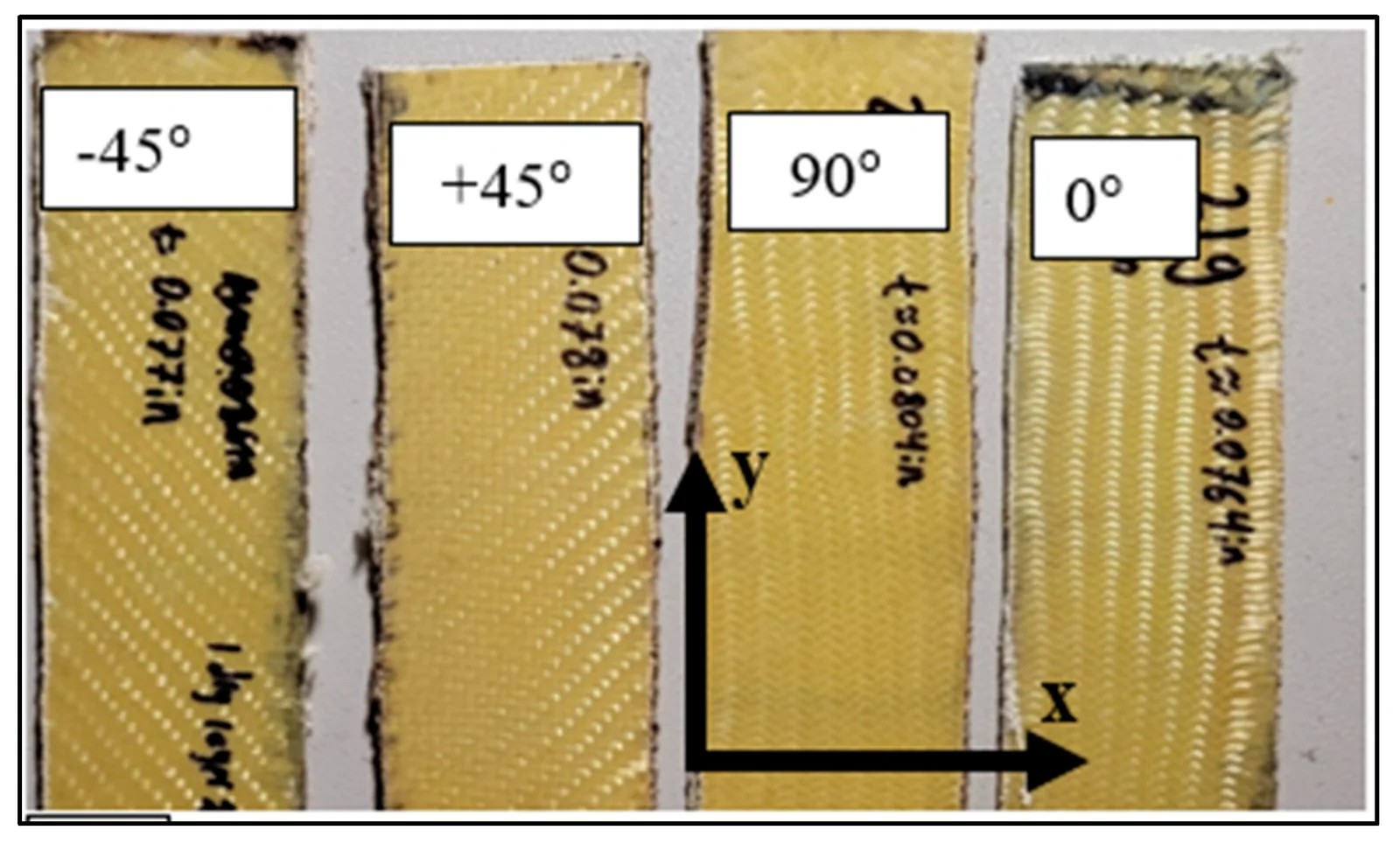
Samples of laminates with fiber orientations of 0°, 45°, and 90°.

One fundamental test used for characterizing the mechanical behavior of composites is the tensile test, conducted in accordance with the Standards [23,24], as shown in Figure 9. For aramid–epoxy laminates, it is essential to consider the orientation of fibers relative to the loading axis. Test specimens are prepared as both unidirectional and multidirectional laminates, with fiber orientations of 0°, 90°, and ±45°, allowing for the assessment of anisotropy in mechanical properties, as shown in Figure 10.

**Figure 11:**
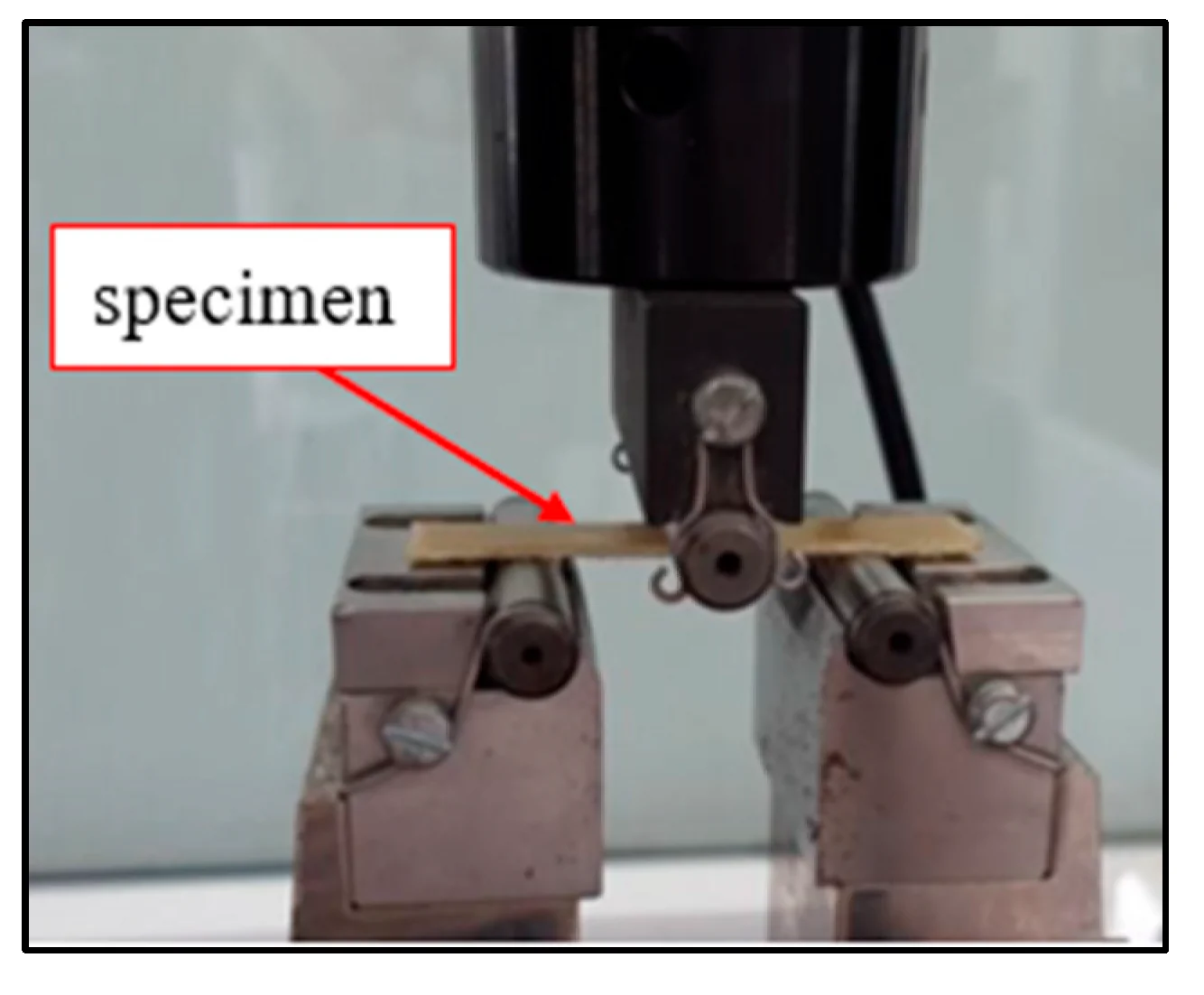
Test setup used for conducting the tensile test with the tested specimen.

Flexural testing constitutes a complementary method used for the tensile testing of polymer composites, enabling the assessment of material behavior under a complex stress state that simultaneously involves tensile and compressive regions within the cross-section. In experimental practice, three- or four-point bending configurations are most commonly employed, in accordance with ASTM D790 [25], allowing for the determination of parameters such as flexural strength, flexural modulus, and the stress–strain response up to failure (Figure 11). In the case of aramid–epoxy laminates, this method is particularly important, as these materials exhibit different resistances to tensile and compressive loading. The failure mechanisms may include matrix cracking, the initiation of delamination, the local instability of fibers in the compressive zone, and progressive interfacial damage.

**Figure 12:**
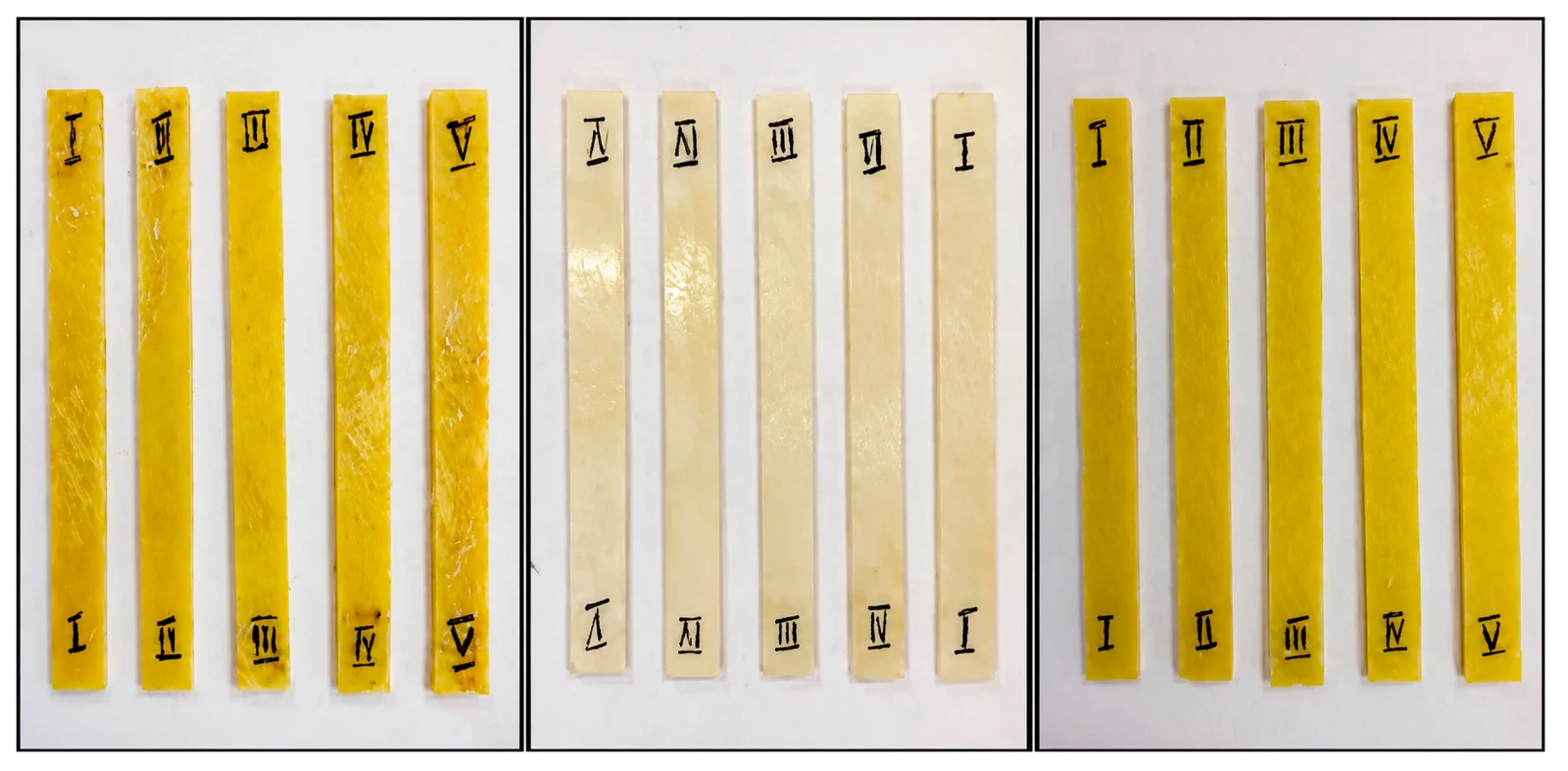
Three Kevlar PVB and epoxy resin specimens used for the 3-point bending test.

Geometric schemes of the three-point bending specimens for 3 examples of Kevlar PVB and epoxy resin are presented in Figure 12, illustrating a beam supported at two points with a centrally applied load. This configuration enables the generation of a maximum bending moment in the middle section of the specimen, where the stress state is dominated by tensile and compressive stresses distributed across the cross-section. The upper surface of the specimen is subjected to compressive stress, while the lower surface experiences tensile stresses, with a neutral axis located between them where the normal stress is equal to zero.

Another key mechanical parameter determining the behavior of layered composites under real service conditions is their shear resistance, particularly within the plane of the laminate. For epoxy composites reinforced with aramid fibers such as Kevlar, shear properties are essential for assessing resistance to delamination, the initiation of interlaminar damage, and the ability to transfer complex loading conditions. One of the most commonly used and well-documented experimental methods for determining the shear properties of composite materials is the Iosipescu method.

**Figure 13:**
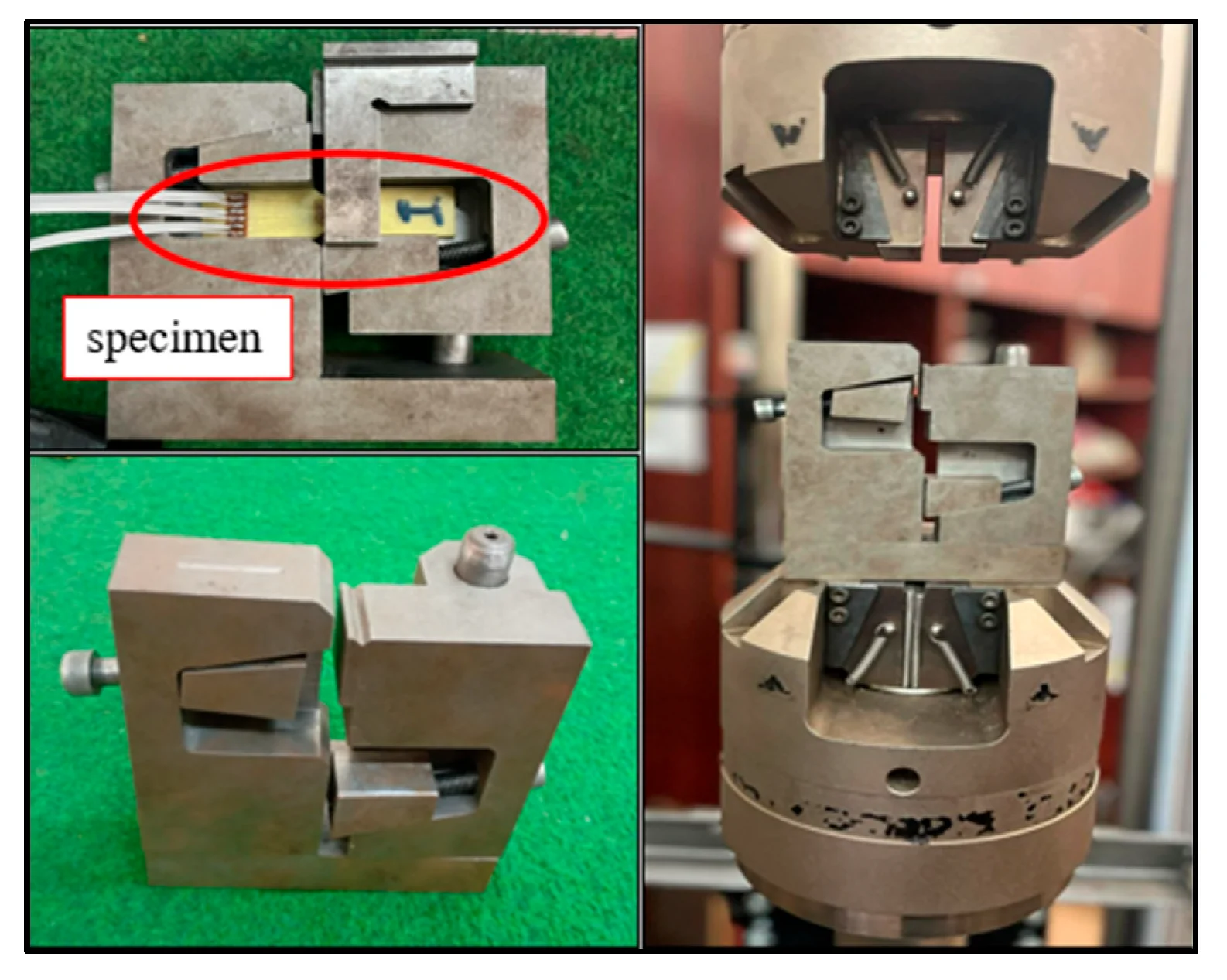
Samples for the 3 examples of Kevlar PVB and epoxy resin used for 3-point bending test.

**Figure 14:**
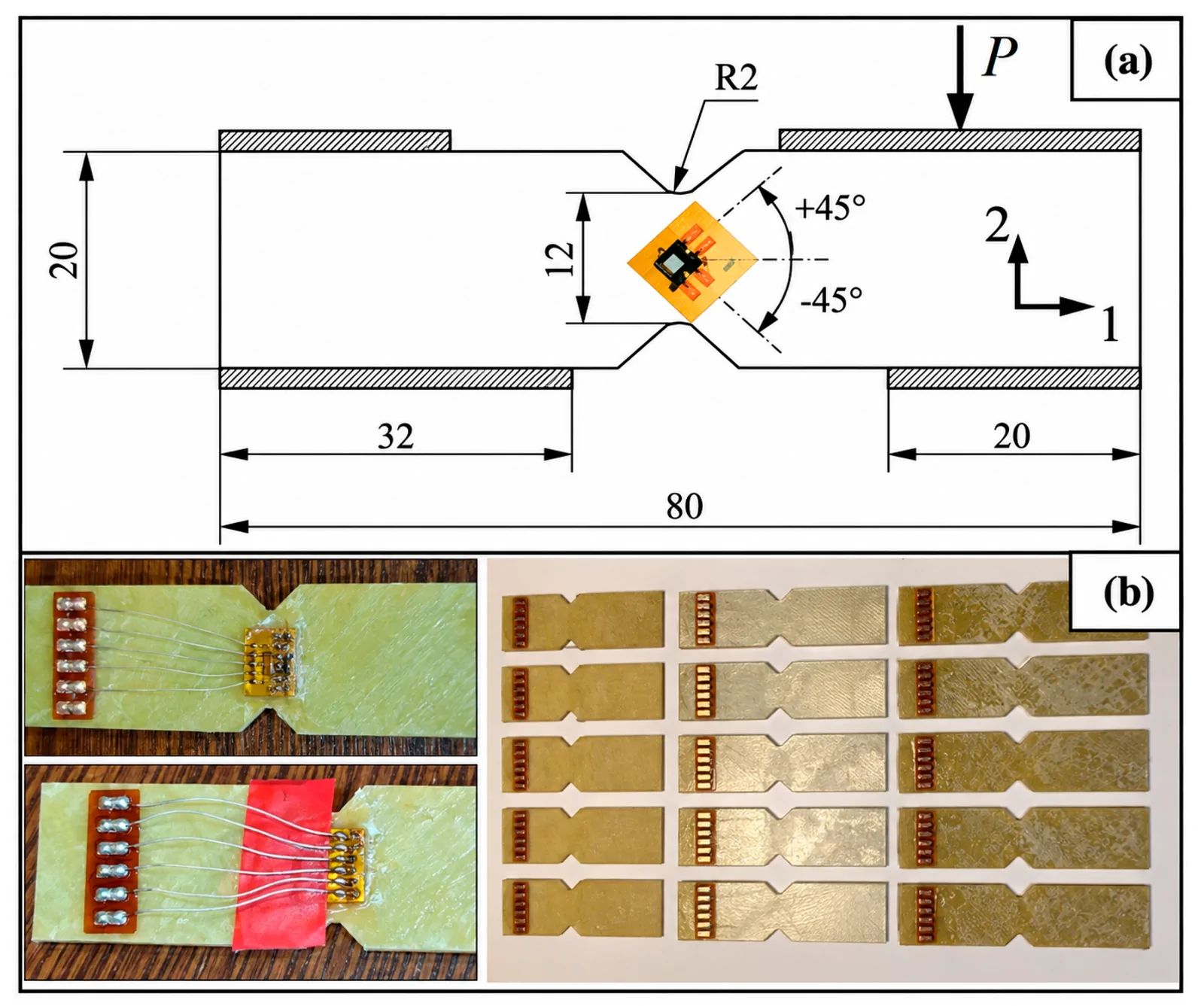
Test setup used for conducting the share test with the: (**a**) tested specimen; (**b**) own samples.

The Iosipescu method, standardized in standards [24], enables the determination of shear strength and shear modulus under a relatively uniform stress state within the measurement region of the specimen. In contrast to conventional tensile or flexural tests, this method allows for the direct evaluation of material behavior under a dominant shear stress state, which is particularly important for laminates with ±45° fiber orientations and for analyzing interfacial behavior in fiber-reinforced composites. Figure 13 presents the experimental setup used for the Iosipescu method, in which the specimen is mounted between specially designed loading fixtures. Specimens prepared via machining for testing are shown in Figure 14.

The geometric scheme of the Iosipescu specimen is presented in Figure 14, illustrating the characteristic double V-notch configuration with notch angles of ±45°, whose purpose is to localize the shear stress state within the central region of the specimen. Additionally, a strain gauge is mounted on the specimen to measure deformation, as the recorded change in electrical resistance is proportional to the strain and enables the determination of stress.

In the case of Kevlar–epoxy composites, the Iosipescu method allows for performing a detailed analysis of the influence of laminate architecture, fiber orientation, and the quality of adhesion at the fiber–matrix interface on shear properties. As reported in [26,27], the shear behavior of aramid laminates is particularly sensitive to interlaminar defects and insufficient fiber impregnation, which makes this method highly effective for assessing the quality of the manufactured material.

## 3. Results and Discussion

Mechanical properties are a fundamental criterion for assessing both the quality and applicability of composite materials in structural engineering. For epoxy composites reinforced with aramid fibers, such as Kevlar, particular emphasis is placed on parameters including governing load-bearing capacity, stiffness, and resistance to mechanical damage. Among the most critical are tensile and flexural strength, compressive and shear properties, and impact resistance, delamination resistance, and the capability to sustain complex loading conditions during service. The comprehensive characterization of these properties provides essential input data not only for the design of engineering structures but also for the advanced numerical modeling and structural validation of composite systems [28].

The mechanical tests were selected to provide a comprehensive evaluation of the composite’s performance under different loading conditions that were representative of potential service applications. Tensile testing was performed to determine the material’s tensile strength, elastic modulus, and elongation at break, fundamental indicators of its load-bearing capacity under uniaxial tension.

Flexural testing was chosen because composite materials are frequently subjected to bending loads in practical applications. This test provides information on flexural strength and flexural modulus, reflecting the combined effects of tensile and compressive stresses within the material.

Shear testing was included to evaluate interfacial bonding between the fibers and the matrix, as well as the material’s resistance to shear deformation. Shear properties are particularly important for fiber-reinforced composites because failure often initiates at the fiber–matrix interface under shear loading conditions.

**Figure 15:**
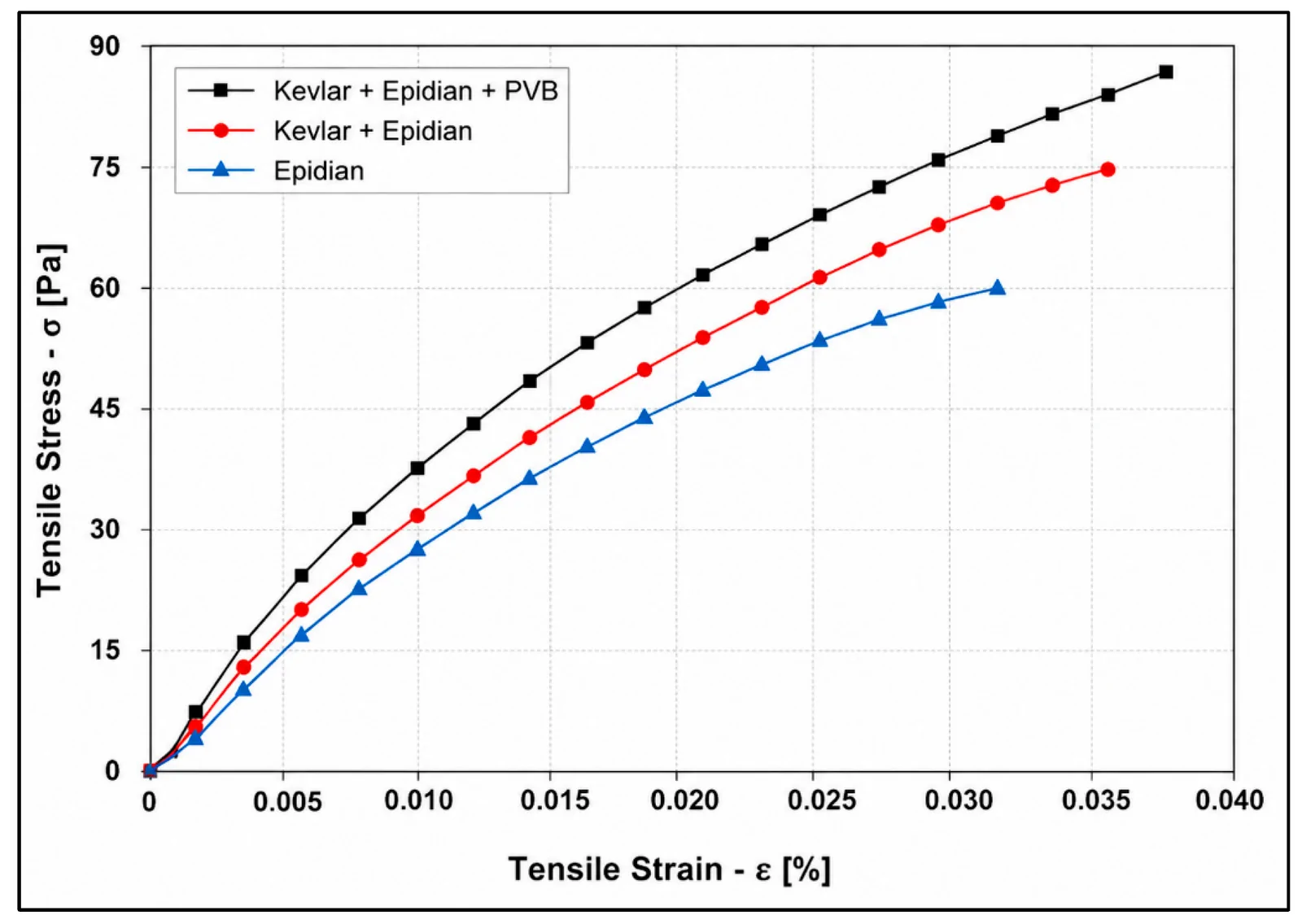
Stress–strain curves used for the tensile testing of composites (chart based on data presented in [27,29]).

In previously published articles, one of the primary experimental methods used to characterize the mechanical response of composite materials is the tensile test, performed in accordance with ASTM D3039 [23]. This test enables the determination of key mechanical parameters, including tensile strength, Young’s modulus, and the complete stress–strain response, as illustrated in Figure 15. For aramid–epoxy laminates, it is imperative to account for the orientation of fibers with respect to the loading direction, due to the inherently anisotropic nature of such materials. Accordingly, test specimens are prepared as both unidirectional and multidirectional laminates, typically with fiber orientations of 0°, 90°, and ±45°, allowing for the systematic evaluation of anisotropy in mechanical behavior.

The experimental investigations conducted clearly demonstrate the significant influence of both the presence of aramid fibers and the application of a PVB coating on the enhancement of the mechanical properties of the material. The most favorable results were obtained for the Kevlar + Epidian + PVB composite, which exhibits the highest stress values across the entire strain range compared to the other tested materials. Our research shows that the maximum stress achieved for this variant reaches approximately 80–85 MPa, representing a substantial increase relative to the Kevlar + Epidian composite, for which the corresponding values are 65–70 MPa. In contrast, the neat epoxy resin exhibits significantly lower maximum stress values, not exceeding 55 MPa, as presented in Figure 15.

The analysis of the stress–strain curves further indicates that incorporating a PVB coating not only enhances tensile strength but also improves the initial stiffness and the material’s ability to sustain higher loads at increased strain levels. The curve corresponding to the composite with the PVB coating is characterized by a more stable response and a delayed onset of damage compared to the system without this interlayer. These findings confirm the effectiveness of interfacial modification carried out through polymer coatings and highlight the considerable potential of epoxy composites reinforced with recycled Kevlar and modified with a PVB layer as advanced structural materials with improved mechanical performance, as previously highlighted in [28].

A key factor determining the mechanical performance of long-fiber-reinforced composites is the orientation of fibers relative to the direction of the applied load. For epoxy composites reinforced with aramid fibers such as Kevlar, variation in fiber orientation leads to pronounced differences in both load-bearing capacity and failure mechanisms. Analysis of the stress–strain curves presented in Figure 15 clearly indicates that changes in fiber orientation result in significant differences in the achieved stress levels, as well as in the deformation characteristics and failure mechanisms of the laminates. For laminates with a 0° fiber orientation, i.e., fibers aligned parallel to the loading direction, the highest stress values are obtained.

**Figure 16:**
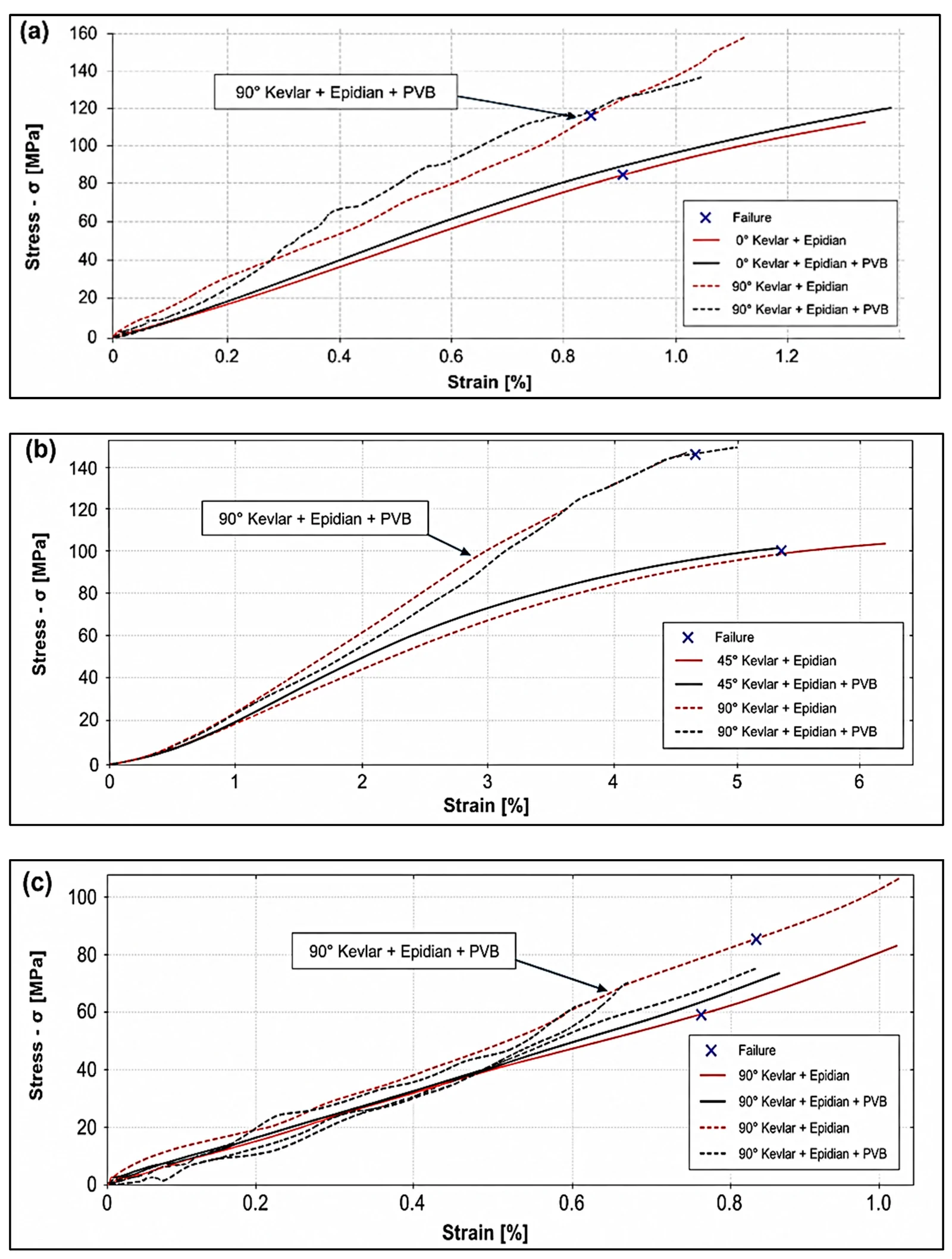
Stress–strain curves (**a**) for 0° fiber orientation, (**b**) for 45° fiber orientation, and (**c**) for 90° fiber orientation; this figure was adapted from (charts developed on the data presented in [12]).

As shown in Figure 16a, the Kevlar + Epidian composite achieves maximum stress levels of approximately 120–130 MPa at strains of about 0.8–0.9%. Introducing an additional PVB interlayer further enhances the load-bearing capacity, with maximum stresses increasing to approximately 145–155 MPa, accompanied by an increase in strain beyond 1.0%. The shapes of the stress–strain curves reflect high initial stiffness and efficient load transfer through the aramid fibers, which, in this configuration, carry the dominant portion of the applied load.

For a 45° fiber orientation, a markedly different mechanical response is observed, associated with a significant contribution of in-plane shear stresses. As indicated in Figure 16b, the maximum stress for the Kevlar + Epidian composite ranges between approximately 90 and 100 MPa, while the application of a PVB coating increases this value to between approximately 130 and 140 MPa. At the same time, laminates with a 45° orientation exhibit substantially higher failure strains, achieving values of up to 4–5%, indicating an enhanced ability to redistribute stresses and absorb deformation energy. The σ–ε curves display a more nonlinear character, reflecting progressive matrix degradation and the gradual activation of shear and delamination mechanisms.

The lowest strength values are observed for laminates with a 90° fiber orientation, in which the fibers are arranged perpendicular to the loading direction. In this configuration, the load is primarily carried by the matrix and the fiber–matrix interface, resulting in significantly reduced mechanical performance and earlier onset of failure.

The stress–strain curves show that for the Kevlar + Epidian composite, the maximum stress values do not exceed approximately 60–70 MPa at strains in the order of 0.6–0.7%. The application of a PVB interlayer leads to a moderate improvement in mechanical performance, with maximum stress increasing to approximately 85–95 MPa and the strain range extending to values close to 0.9–1.0%. In this configuration, the load is primarily carried by the epoxy matrix and the interfacial layer, while the contribution of the fibers is largely limited to the structural stabilization of the laminate.

PVB increases the tensile strength at break because it improves the adhesion between the fibers and the matrix, allowing stresses to be transferred more efficiently. It also enhances the material’s toughness by delaying crack initiation and propagation, enabling the composite to withstand higher loads before failure.

Some fiber orientations are more effective than others because the mechanical properties of fiber-reinforced composites are highly anisotropic. Fibers aligned with the loading direction carry the applied load more efficiently, resulting in higher tensile strength and stiffness. In contrast, fibers oriented at angles to the loading direction contribute less to load-bearing and are more susceptible to matrix-dominated failure mechanisms, leading to lower mechanical performance.

A comparative analysis of all fiber orientations clearly indicates that the highest strength and stiffness are achieved for the 0° configuration, whereas the 45° orientation provides a favorable balance between load-bearing capacity and deformability. In contrast, the 90° orientation exhibits the lowest strength; however, its mechanical performance can be significantly improved through the application of PVB interlayers.

The obtained results confirm that appropriately selecting the fiber orientation, together with interfacial modification strategies, constitutes an effective approach for tailoring the mechanical properties of aramid–epoxy composites. This is vital for the design of laminated structures with specified strength and service performance requirements.

Flexural testing constitutes a complementary method for the tensile testing of polymer composites, enabling the evaluation of material behavior under a complex stress state that simultaneously involves tensile and compressive regions within the cross-section.

For aramid–epoxy laminates, this method is particularly important, as these materials exhibit different resistances to tensile and compressive loading. The associated failure mechanisms may include matrix cracking, the initiation of delamination, the local instability of fibers in the compressive zone, and progressive interfacial damage [28]. Figure 16 presents the stress–strain curves for two material systems: Kevlar + Epidian and Kevlar + Epidian + PVB. Analyses of the curves clearly indicate that the incorporation of an intermediate PVB layer–adhesive coating significantly improves flexural load-bearing capacity across the entire strain range compared to the laminate without this modification.

**Figure 17:**
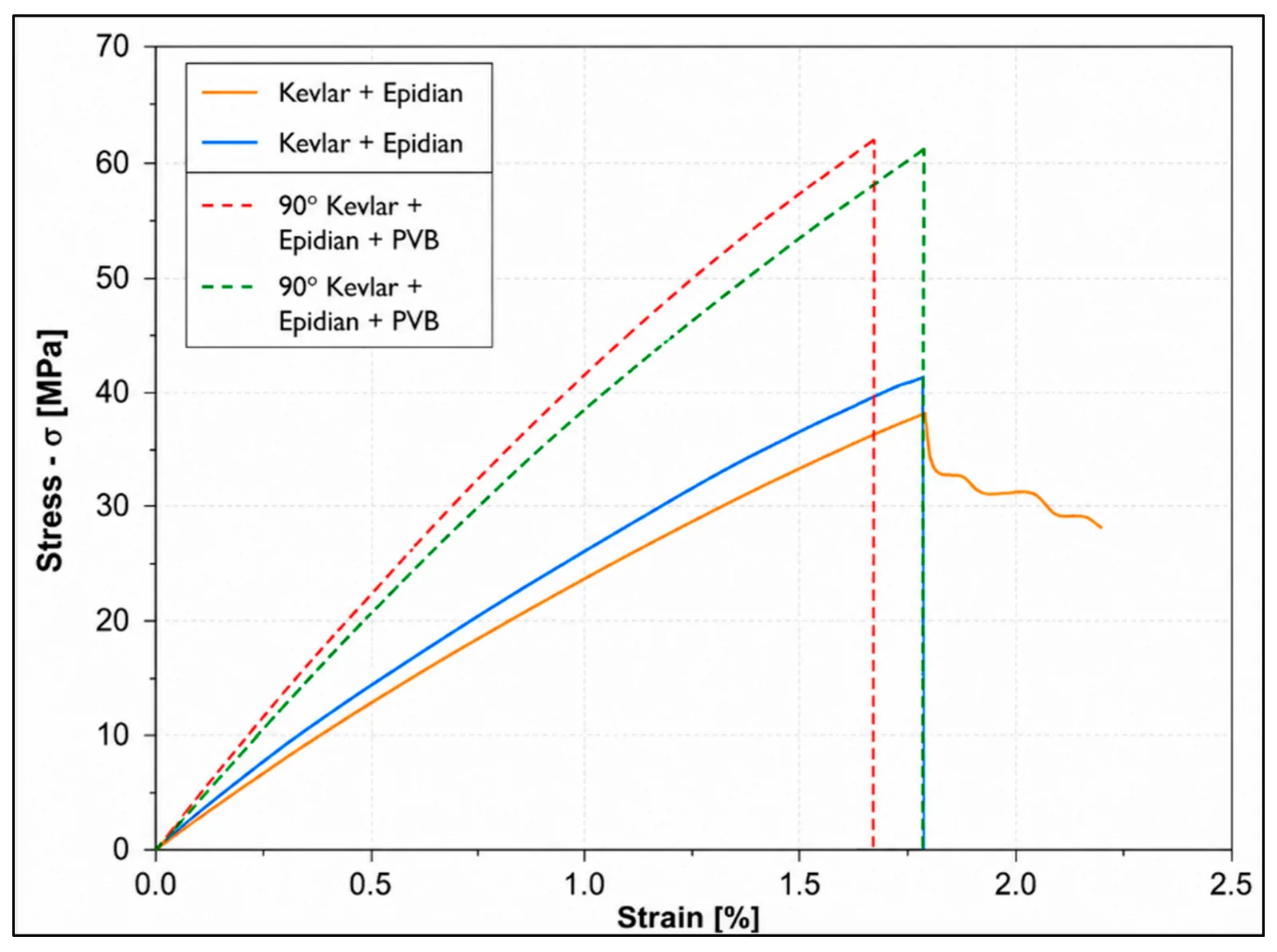
Characteristics of stress–strain curves in three-point bending tests for two different materials (chart developed on the data presented in [28]).

For the Kevlar + Epidian laminate, the maximum flexural stress is approximately 40 MPa at a strain level of about 1.7% is presented on Figure 17. Within this range, a distinct critical point is observed, followed by a rapid decrease in stress, characteristic of damage initiation and propagation mechanisms, such as matrix cracking or local delamination. In the post-critical region, the curves exhibit a limited capacity for further load transfer, indicating a rapid transition to failure with a predominantly brittle character and minimal contribution from stabilizing mechanisms. The observed strengthening effect can be interpreted thanks to improved interaction between phases at the fiber–matrix interface, achieved through the application of PVB as an intermediate layer.

Under bending conditions, stress concentrations are particularly significant in the vicinity of microstructural discontinuities, where matrix microcracking and delamination are initiated. The PVB layer, due to its more compliant and viscoelastic character, can reduce local stress concentrations, enhance the wetting of aramid fiber surfaces by the epoxy matrix, and increase resistance to debonding and delamination. Consequently, the laminate exhibits an increased ability to transfer bending loads up to the point of critical damage initiation.

The results of the flexural tests confirm that interfacial modification using PVB is an effective approach for improving the mechanical performance of Kevlar–epoxy composites, particularly under loading conditions dominated by delamination and matrix cracking mechanisms. From the perspective of structural material design, the obtained maximum stress values and the shape of the σ–ε curves indicate the potential to increase the load-bearing capacity of laminated components while maintaining a comparable range of failure strains. This is particularly advantageous in applications requiring both high strength and an adequate safety margin prior to failure.

**Figure 18:**
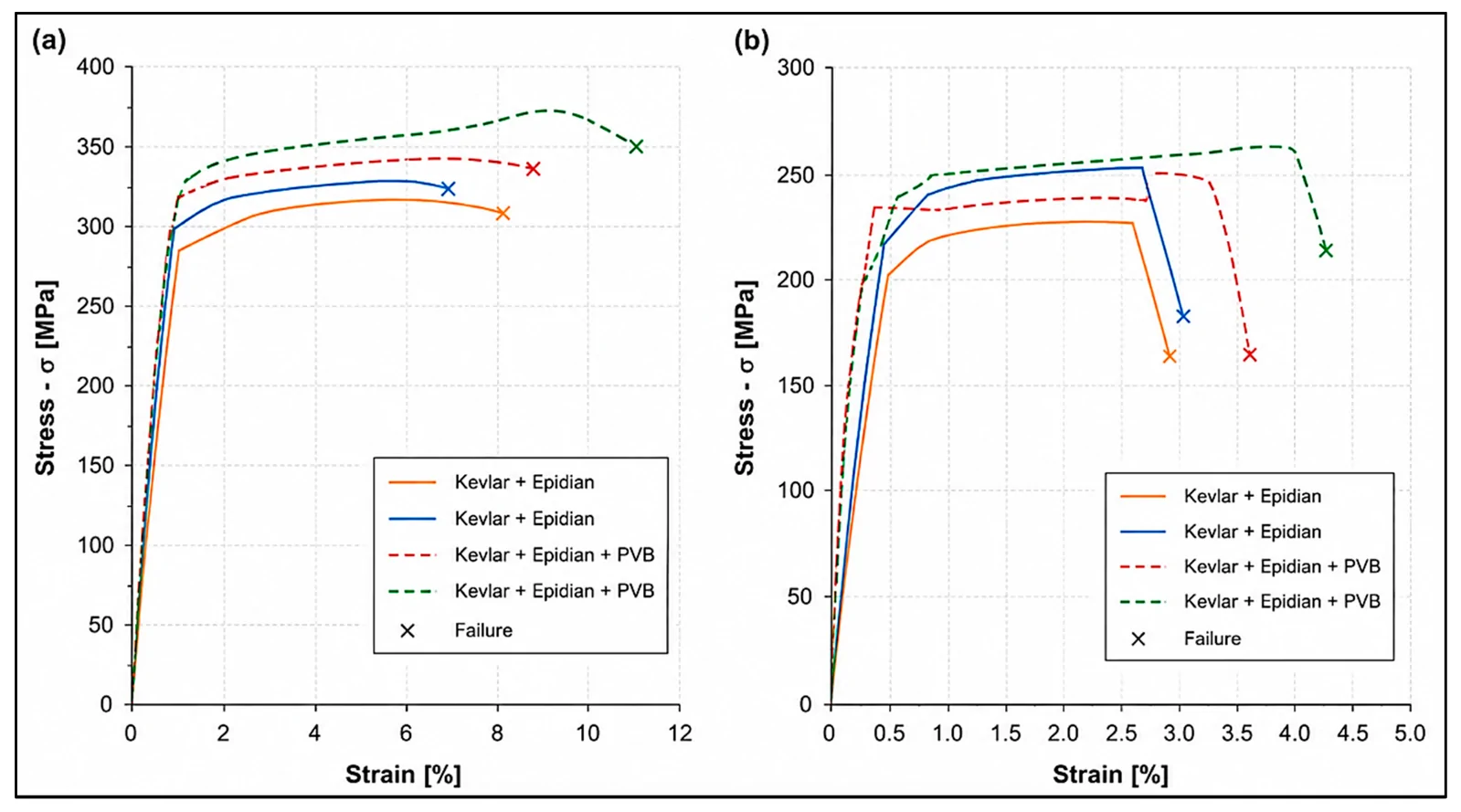
Characteristics of the two variant materials; stress–strain curves: (chart based on data presented in [30]). (**a**) Kevlar + PVB; (**b**) Kevlar.

In [28], both force–displacement and stress–strain curves were recorded during shear testing using the Iosipescu method. Analysis of the force–displacement characteristics presented in Figure 18a indicates that all investigated material systems exhibit a similar initial response, characterized by a rapid increase in force within the elastic range, followed by a transition to a plateau region corresponding to progressive material degradation.

However, clear differences between the individual material variants are observed for maximum force values and displacement at failure. For the Kevlar–epoxy–PVB composite, the highest maximum force values were obtained, with values of approximately 350 MPa for variant 1 and about 325 MPa for variant 2. At the same time, the largest displacements at failure, in the order of 12 mm, were recorded for variant 1.

The shape of the curve indicates a relatively extended phase of residual load-bearing capacity, suggesting a gradual degradation process and the involvement of mechanisms such as interlaminar friction and partial fiber bridging by aramid fibers. In contrast, for composites without the PVB interlayer, the maximum force values were slightly lower, i.e., approximately 12 kN. The displacement failure for these materials was about 7.8 mm, indicating earlier damage initiation and more rapid propagation, ultimately leading to premature failure of the specimen.

As reported in [30], more pronounced differences between the investigated materials are observed in the analysis of shear stress–strain curves (Figure 18b). For the Kevlar–epoxy composite, the maximum shear stress was approximately 275–282 MPa, with failure strains of 4.0–4.5%. This represents the highest shear strength among the analyzed variants, confirming the material’s strong capability to transfer in-plane shear loads.

For composites containing a PVB interlayer, the maximum shear stresses were slightly lower, ranging from approximately 261 to 272 MPa, with failure occurring at strains of about 3.0–4%. However, compared to the reference variant, for which the shear stress did not exceed 235–245 MPa and the failure strain was approximately 2.8–3.1%, the incorporation of PVB resulted in clear improvements in both load-bearing capacity and deformation capability.

The observed differences in curve characteristics and limit values can be interpreted in the context of the role of the interfacial region in aramid–epoxy composites. Kevlar fibers, due to their chemical structure, exhibit limited adhesion to conventional epoxy matrices, promoting fiber–matrix debonding and matrix cracking under shear loading. The introduction of a PVB interlayer acting as an intermediate phase improves fiber wettability and reduces local stress concentrations at the interface.

As a result, a delay in the initiation of dominant failure mechanisms is observed, along with increased resistance to progressive damage. This is reflected in higher achievable shear stresses and greater failure strains, confirming the beneficial effect of interfacial modification on the mechanical performance of the composite.

To minimize the influence of specimen-to-specimen variability, the results were averaged across replicate samples tested under identical conditions. Where applicable, the data were normalized with respect to specimen dimensions (e.g., the cross-sectional area) to enable direct comparison between samples. No additional scaling or transformation of the data was applied unless otherwise specified. The processed data were then used to generate the figures and statistical comparisons presented in this study.

## 4. Conclusions

In summary, for research on novel composite materials, PVB waste can serve as either a flexible polymer matrix or a modifying component influencing the mechanical properties of multiphase systems. Its high deformability, strong adhesion to various materials, and effective damping characteristics make it particularly attractive for applications requiring energy absorption, vibration reduction, and enhanced resistance to impact loading.

Moreover, the incorporation of PVB waste into composite structures aligns with the principles of the circular economy, enabling reduced waste streams and the rational utilization of polymer resources, while simultaneously facilitating the development of materials with new and functionally advantageous properties. The outlook for the market in personal protective equipment based on Kevlar appears highly promising, with numerous trends and growth projections indicating dynamic development through 2030. The convergence of increasing safety awareness, increasing concerns over security, ongoing technological advancements, and the growing emphasis on sustainability and environmental responsibility is expected to drive further expansion in this sector.

An increase in the mass fraction of long fibers in composite materials improves their elastic properties; however, this effect is governed by several factors, including the quality of interfacial adhesion, structural homogeneity, fiber orientation, and the technological capabilities of the manufacturing process. These relationships are vital both in the design of new composite materials and in the analysis of their mechanical behavior in structural applications.

Tensile tests demonstrated that the highest ultimate stress values and elastic moduli are achieved for laminates with a 0° fiber orientation, in which the load is predominantly carried by the aramid fibers. Laminates with a ±45° orientation exhibited increased deformability, while 90° configurations showed the lowest strength, governed primarily by the properties of the matrix. The incorporation of a PVB coating resulted in an increase in maximum tensile stresses, as well as higher strain at failure, indicating improved fiber–matrix adhesion and a more stable degradation process.

Flexural tests provided an important complement to tensile experiments, enabling the evaluation of laminate resistance to bending, delamination initiation, and brittle fracture. The results confirmed that Kevlar–epoxy composites exhibit higher load-bearing capacity and improved ability to sustain bending loads compared to unmodified variants. Moreover, the presence of a PVB interlayer contributed to a more gradual failure process and increased energy absorption prior to fracture.

Shear properties were evaluated using the Iosipescu method, allowing for the clear identification of damage mechanisms occurring within the interfacial region. The highest shear stress values were obtained for Kevlar–epoxy composites, accompanied by relatively large failure strains, indicating high resistance to degradation under complex stress states. The introduction of a PVB interlayer further enhanced deformation capability and resulted in a more stable stress–strain response, confirming the beneficial effect of interfacial modification on shear resistance and delamination behavior.

The conducted experimental investigations of the mechanical properties of epoxy composites reinforced with aramid fibers (Kevlar) enabled a comprehensive assessment of their behavior under fundamental loading conditions, namely tension, bending, and shear. Analysis of the obtained results clearly confirmed the significant influence of both fiber orientation and interfacial modification through the application of a PVB interlayer on the load-bearing capacity, stiffness, and failure mechanisms of the investigated materials.

It should be emphasized that, depending on the anticipated service conditions and structural applications of the composites, the scope of experimental investigations may be extended to include additional tests, such as compression, impact resistance, and fatigue analysis. Compression tests enable the evaluation of susceptibility to fiber buckling and matrix degradation, which often govern the load-bearing capacity of thin-walled structures. Therefore, a comprehensive experimental approach, encompassing multiple loading conditions, constitutes an essential foundation for the reliable design and numerical modeling of aramid–epoxy composites with tailored mechanical properties.

In future studies, researchers should focus on optimizing PVB content and processing conditions to further improve interfacial bonding and overall composite performance. Additional studies on long-term durability, fatigue behavior, moisture resistance, thermal stability, and impact resistance are also recommended to evaluate the suitability of these composites for use in demanding service environments. Furthermore, investigating different fiber architectures and surface treatments could provide additional improvements in mechanical properties and expand the range of potential industrial applications.

## Data Availability

No new data were created or analyzed in this study. Data sharing is not applicable to this article.
